# The first and second zinc finger domains from Poly-ADP-ribose polymerase 1 (PARP1) are modified by hydrogen sulfide

**DOI:** 10.1007/s00775-026-02134-3

**Published:** 2026-05-22

**Authors:** Ayanna J. Williams, Sarah L. J. Michel

**Affiliations:** Department of Pharmaceutical Sciences, University of Maryland Baltimore School of Pharmacy, Baltimore, MD 21201 USA

**Keywords:** Zinc-finger, Metalloproteins, PARP-1, Hydrogen sulfide, Persulfidation

## Abstract

Poly(ADP-ribose) polymerase 1 (PARP1) is a Zinc Finger (ZF) protein that is involved in DNA damage response. PARP1 contains three zinc finger (ZF) domains, two with a Cys_2_HisCys (CCHC) ligand set and one with a Cys_4_ (CCCC) ligand set that coordinate zinc. Persulfidation of cysteine residues in proteins is a new type of post-translational modification (PTM), for which ZFs are emerging as frequently modified. PARP1 has been identified as persulfidated in several cellular based persulfide-specific proteomics studies across several different cell types. Here, we examine PARP1 persulfidation by H_2_S. Two PARP1 peptide constructs, shPARP1-ZF1 and shPARP1-ZF2, were synthesized, purified, and shown to bind zinc and fold, using Co(II)/Zn(II) UV-visible (UV) monitored titrations and circular dichroism (CD) spectroscopy. The reactivity of the PARP ZFs with H_2_S was assessed using a UV-visible monitored 4-chloro-7-nitrobenzofurazan (NBF-Cl)/dimedone switch-tagging approach. Persulfidation for both PARP1 peptide constructs was observed under zinc-bound, oxygenated conditions. Superoxide intermediates were detected during the reaction using both dihydroethidine fluorescence and superoxide dismutase trapping. Together, these findings provide evidence that CCHC-type PARP ZFs can undergo persulfidation when Zn is bound and provide support for persulfidation being a general post-translational (PTM) modification for ZFs with different secondary structural elements.

**Graphical abstract:**

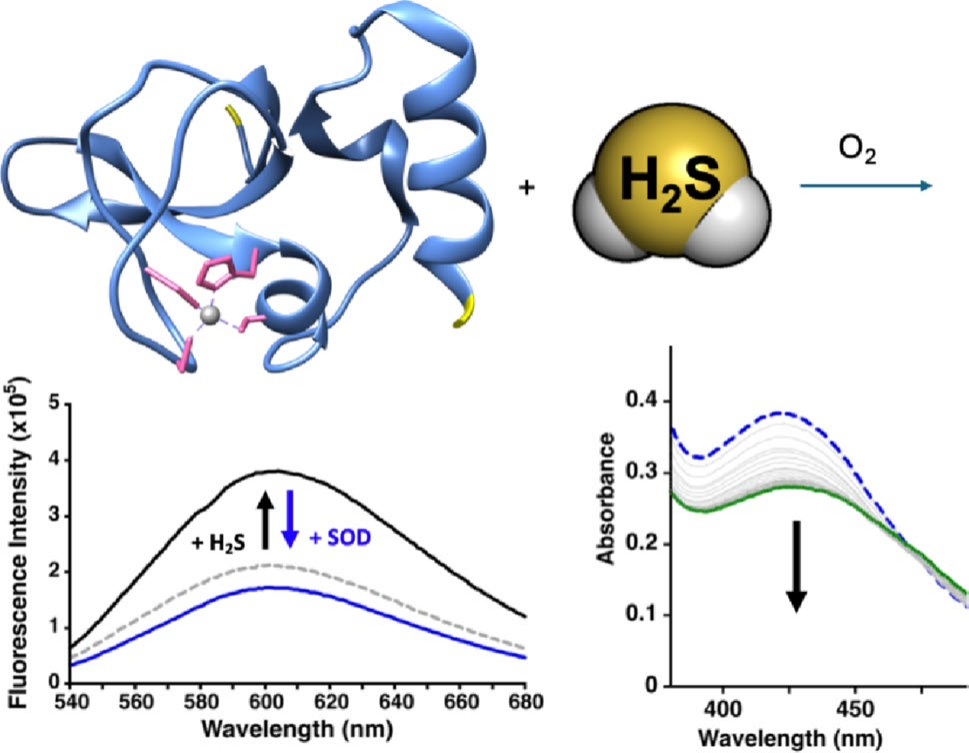

**Supplementary Information:**

The online version contains supplementary material available at 10.1007/s00775-026-02134-3.

Zinc Finger proteins (ZFs) are a large class of primarily eukaryotic proteins that use Zn as a structural co-factor [1–4]. All ZFs contain domains with repeats of four cysteine (cys, C) and/or histidine (his, H) residues. These residues serve as the Zn coordinating ligands, to which Zn binds in a tetrahedral geometry. The number of cysteine versus histidine ligands and the spacing between the cysteine and histidine ligands vary between ZFs [2–4]. Typical ligand sets include CCHH, CCCH, and CCHC and CCCC, and ZFs are often classified based upon these ligand sets. ZFs have a variety of biological functions including the regulation of gene expression, cell proliferation, signal transduction, and apoptosis [1, 2, 5, 6] .

A common feature of ZFs is that they are unstructured in the absence of Zn, and adopt secondary structure when Zn is coordinated. Thus, the paradigm for ZFs is that they are structural - zinc binding leads to folding [7] which leads to function [1, 3, 4, 8–11]. More recently, there have been reports that ZFs can be modified, exogenously, as a means to modulate function [12–14]. This includes work from our laboratory in which we reported that ZF protein, tristetraprolin (TTP) can be modified at its cysteine residues to form persulfides by the gaseous signaling molecule hydrogen sulfide (H_2_S) [1, 15–17].

H_2_S is a gasotransmitter, the third after CO and NO. H_2_S is involved in the regulation of a range of physiological processes including vasodilation, neuromodulation, cardiovascular function, metabolism, apoptosis, and inflammation [18–28]. How H_2_S regulates these cellular functions is still being unraveled. At the molecular level, H_2_S has been shown to react with heme proteins, with reactive oxygen and nitrogen species, and with cysteine residues to form persulfides [29–33]. The modification of cysteine residues to persulfides (RSH to RSSH) is a post-translational modification (PTM) that has been proposed to be as important as phosphorylation [33, 34].

The proteins that are persulfidated by H_2_S are not well defined. H_2_S does not directly react with cysteine thiols, as H_2_S is predominantly HS^−^ at physiological pH owing to a pKa of 6.8 [33]. There are several reports of persulfidation of ZF proteins, including Parkin, MYND, Sirtuin, and the androgen receptor [35–39]. How cysteine residues of ZFs are persulfidated was not clear from these studies. Our laboratory examined the ZF persulfidation reaction at the molecular level. Our work focussed on tristetraprolin (TTP), which is a CCCH type ZF protein that regulates mRNA processing of cytokines and chemokines [1, 16, 40]. When the protein is loaded with Zn, each of the two ZF domains adopts secondary structure that allows for RNA binding in vitro and mRNA regulation in cells [1, 16, 40, 41]. We reported that when a construct of Zn(II)-TTP that contains the two ZF domains is reacted with H_2_S, in the presence of O_2_, persulfidated intermediates are formed. The collective data from this work supported a model for ZF persulfidation in which the zinc center acts as a conduit for electron transfer between HS^−^ and O_2_ (scheme S1) [15]. We then investigated how ligand set impacted persulfidation of ZFs by preparing a series of mutants of the TTP ZFs in which the ligand set was modified to CCCC, CCHH and CHHH and investigating their reactivity with H_2_S [17]. The TTP mutants had different ligand sets but the same overall fold, and we determined that all variants were persulfidated by H_2_S under the same conditions as wild type TTP – when Zn was coordinated and O_2_ was present [17]. Additionally, a synthetic N_2_S-Zn complex exhibited the same persulfidation chemistry, providing further support for the proposed mechanism [17]. ZFs are highly abundant and varied in eukaryotes, and these collective data led us to ask a broader question: is persulfidation of ZF proteins a common PTM? We employed persulfide-specific proteomics in MEF cells along with a comprehensive meta-analysis of all published persulfide-selective proteomic data to address this question. This work revealed that ZF persulfidation is widespread in cells from different types of eukaryotes. We found that it occurs in ZFs of all classes, with CCCC, CCHC, CCCH and CCHH ligand sets, and with different folds [17, 42].

ZFs vary with respect to their overall fold and function, in addition to ligand set, and it is not yet known whether persulfidation of ZFs with secondary structure that is different from TTP occurs in the same Zn and O_2_ dependent manner. Here we sought to examine ZF persulfidation of a ZF with different secondary structure. We examined the persulfide specific proteomics data that we had analyzed and selected Poly-ADP-ribose polymerase 1 (PARP1) to investigate. This ZF was chosen because it is found in multiple persulfide specific proteomics data sets: there is evidence for PARP1 persulfidation in *homo sapiens*, *rattus norvegicus*, and *mus musculus* [42]. PARP1 is involved in DNA repair, genomic stability and gene regulation [43]. The architecture of PARP1 includes three ZF domains at the n-terminus, a central BRCT auto-modification domain, and a c-terminal WGR domain, Fig. 1 [44, 45]. The three ZF domains differ with regards to ligand set and fold - ZF1 and ZF2 are CCHC-type ZFs adopt a ββα structure upon Zn^2+^ coordination [44, 46–48] while ZF3 is a CCCC type ZF, that adopts a ‘zinc ribbon’ type fold when Zn^2+^ is bound, Fig. 1b [46, 48–52]. These folds are different from that of TTP, which forms a structure dominated by loops and turns when Zn is bound [2].

**Fig. 1:**
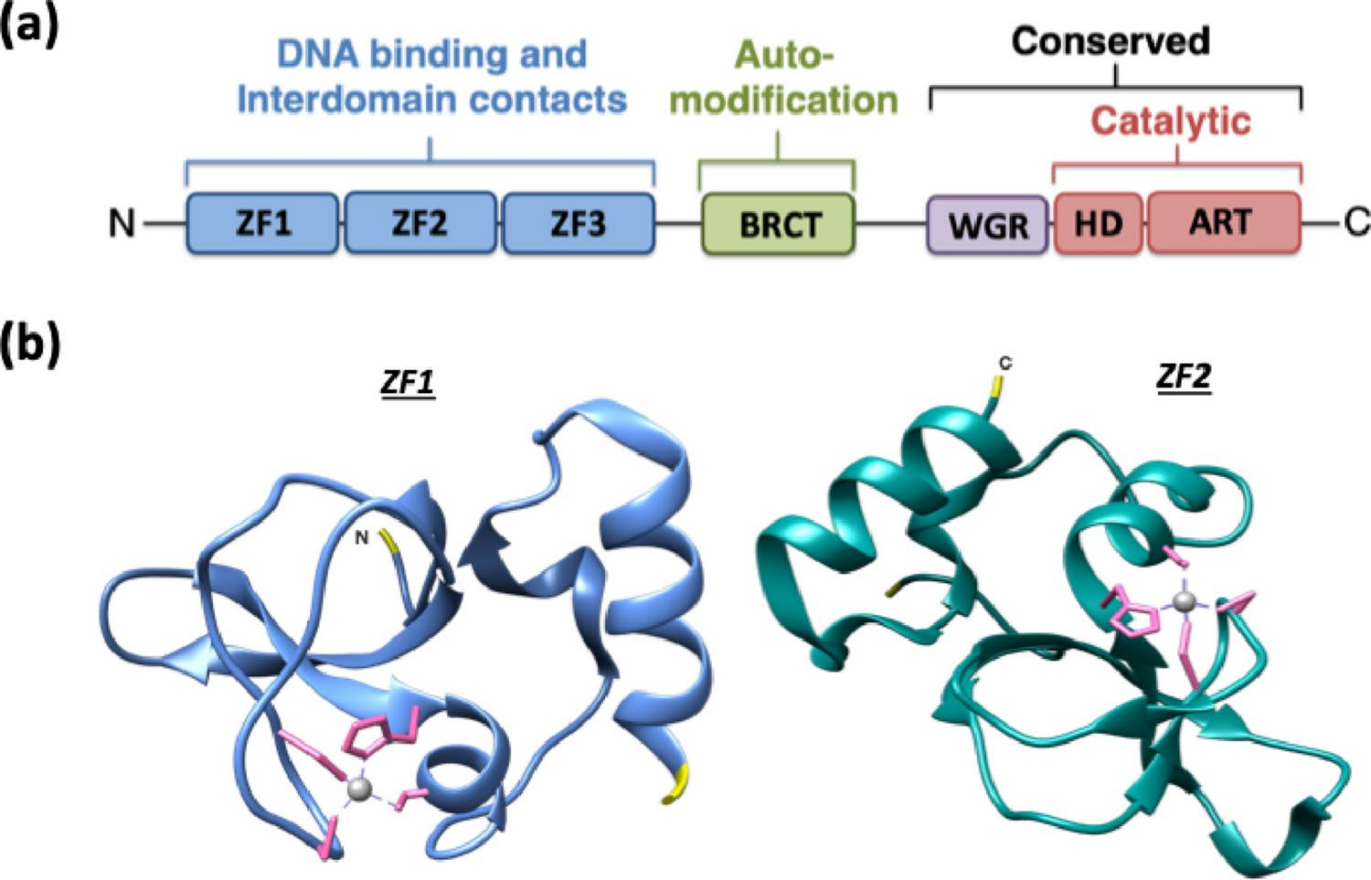
**(a)** Cartoon diagram of PARP1 protein with domains indicated, zinc finger (ZF)s are colored blue. **(b)** X-ray structures of PARP1-ZF1 and PARP1-ZF2, Zn is colored gray, cysteine and histidine ligands are pink. Structure made in Pymol, (PDB: 4AV1)

Herein, we examine the persulfidation of two ZF domains of PARP1, ZF1 and ZF2, by H_2_S. We chose to study these domains, not only because their structures are different than TTP, but also because there are compelling data that exogenous metals modify PARP1 at the cysteine residues of ZF1 and ZF2, affecting structure and function. We hypothesized that H_2_S would be similarly reactive with these domains. The research on exogenous metals interacting with PARP1 includes a series of studies on the connection between arsenic toxicity and arsenic modification of PARP1. Hudson and Liu have shown that As and related complexes can displace Zn in PARP1, affecting the fold of the domains in vitro and the protein function in cells [53–58]. In addition, they have also reported that As induces iNOS leading to S-nitrosation of cysteine residues in PARP1, which is one of the few example of a nitrosation PTM of a ZF by [59]. Liu has also reported uranium displacement of Zn in PARP1 [60]. Similarly, Casini and co-workers have published a series of studies aimed at understanding how gold complexes can target PARP1 as a means to treat cancer. They have shown that both Au(I) and Au(III) complexes can exchange for Zn and modify the coordination geometry and function of PARP1 ZFs [61–65]. Here we report the characterization and reactivity of the ZF1 and ZF2 domains of PARP1 with H_2_S. We obtained evidence for persulfidation of the ZFs in a reaction that requires O_2_ which is analogous to that observed for TTP. These results establish that persulfidation of the PARP1 ZFs occurs via a conserved O_2_ dependent pathway, supporting a common mechanism for persulfidation of ZFs with different types of secondary structure.

## Materials and methods

### Materials and reagents

Tris(2-carboxyethyl)phosphine hydrochloride (TCEP-HCl) was purchased from Thermo Fisher Scientific. Zinc chloride (ZnCl_2_), 4-(2-hydroxyethyl)−1-piperazineethanesulfonic acid (HEPES), trifluoracetic acid (TFA), acetonitrile (HPLC grade), cobalt chloride hexahydrate (CoCl_2_•6H_2_O), sodium phosphate (Na_2_HPO_4_), 4-chloro-7-nitrobenzofurazan (NBF-Cl), 5,5-dimethylcyclohexane-1,3-dione (dimedone), and Chelex 100 sodium form resin were purchased from Sigma-Aldrich. Sodium sulfide anhydrous (Na_2_S) was bought from Alfa Aesar. Dihydroethidium (DHE) was acquired from Invitrogen.

### General considerations

Metal binding experiments were performed in a COY anaerobic glovebox chamber containing a gas mixture with H_2_ mass fraction of 3% and N_2_ mass fraction of 97%. Water used for buffers, peptide resuspension, and other metal binding reagents was first purified using a Milli-Q IQ 7000 water purification system and Q-POD dispenser equipped with a 0.22 μm filter, then chelex-filtered, and further purified through a Millex-GV 0.22 μm filter purchased from Millipore-Sigma. Next, all solutions were degassed first by bubbling argon gas for approximately 30 min, then subject to vacuum degassing overnight to ensure removal of oxygen. All buffers, water, and materials were equilibrated to the anaerobic conditions of the glovebox prior to use. For experiments that involved addition of O_2_, samples were oxygenated for at least one hour, and experiments were performed on the bench top open to air.

### Synthesis and purification of shPARP1-ZF1 and shPARP1-ZF2 peptides

Peptides corresponding to the first zinc finger motif (called shPARP1-ZF1) [GRASCKKCSESIPKDKVPHWYHFSCFWKV] (residues 17–31, 47–60) and second zinc finger motif (called shPARP1-ZF2) [NRSTCKGCMEKIEKGMIDRWYHPGCFVKN] (121–135, 153–166) of PARP1 were synthesized via Genscript at a purity level greater than 75%. These peptide sequences were designed based upon sequences reported by Liu and Hudson [57], Casini [66] and the related sequence by Bal [67]. For a standard purification, 10–20 mg of peptide was suspended in purified, degassed H_2_O, mixed with 5 mM tris(2-carboxyethyl) phosphine (TCEP), and allowed to stand at room temperature for one hour before being filtered through a 0.22 μm filter. Each solution was applied to a Waters Symmetry300^™^ C18-reverse Prep 5 column using a Waters 1525 High Performance Liquid Chromatography (HPLC) instrument. A water/acetonitrile (H_2_O/CH_3_CN, 0.1% trifluoroacetic acid (TFA) gradient was applied and the elutions corresponding to the purified peptides were collected. HPLC elutions containing purified peptide were transferred to an anaerobic glovebox and lyophilized. The presence and mass of the peptides were confirmed by sodium dodecyl-sulfate polyacrylamide gel electrophoresis (SDS-PAGE) and matrix assisted laser desorption ionization – time of flight (MALDI-TOF) mass spectrometry (shPARP-ZF1: calculated 3455.0, observed 3456.6; shPARP-ZF2: calculated, 3432.0, observed 3433.4). All peptides were stored at −20 °C and all further experiments were performed under anaerobic conditions in glovebox.

### Co(II) and Zn(II) binding to PARP1 peptides

UV-visible spectroscopy was performed using a Cary 60 UV-Vis Spectrophotometer (Agilent) in a Coy anaerobic glovebox with 1 cm pathlength quartz cuvettes (Starna Cells). Before each titration, the lyophilized peptide was resuspended in degassed, chelex-filtered H_2_O and allowed to stand for at least 10 min, after which it was resuspended in degassed buffer of 100 mM HEPES at pH 7.5. The optical spectra of apo-shPARP1-ZF1 and apo shPARP1-ZF2 were measured from 220 to 820 nm, and the peptide concentrations were determined using calculated extinction coefficients of 12,490 M^−1^cm^−1^ and 6990 M^−1^cm^−1^, respectively. For a typical titration, peptide concentrations were between 30 µM to 60 µM. Cobalt and zinc binding were determined spectrophotometrically by monitoring the optical spectrum of each peptide, apo-shPARP1-ZF1 and apo-shPARP-ZF2, as first CoCl_2_ was titrated up to 5 equivalents followed by the addition of ZnCl_2_. The addition of CoCl_2_ led to the appearance of new absorbance bands between 500 and 800 (d-d bands) that increased to saturation. The subsequent addition of ZnCl_2_ up to 5 equivalents led to a decrease in the absorbance bands due to Zn replacing Co at the metal binding sites. To determine dissociation constants (K_d_) for Co binding to shPARP1-ZF1 and shPARP1-ZF2, the measured increase in absorbance at 650 nm and 655 nm for shPARP1-ZF1 and shPARP1-ZF2 respectively was plotted versus the concentration of CoCl_2_ titrated. The data were fit to a 1:1 binding model, from which an upper limit dissociation constant (K_d_) was determined. To determine the dissociation constants (K_d_) for Zn binding, the measured absorbance at 650 nm and 655 nm for shPARP1-ZF1 and shPARP1-ZF2, respectively, was fit to a competitive binding model of Berg and Merkle [68]. All experiments were performed in triplicate.

### Secondary structure of PARP1 peptides via circular dichroism (CD) spectroscopy

Circular dichroism spectra for shPARP1-ZF1 and shPARP1-ZF2 were measured between 190 and 250 nm using a Jasco-1500 spectropolarimeter set for high sensitivity using rectangular 1 mm Starna Cell Spectrosil Far UV Quartz cuvettes. A total of five scans were collected and displayed as an average for the final plot that was corrected for buffer blank. Samples were prepared under anaerobic conditions to prevent any oxidation and precipitation of apo-PARP1 peptides. First a buffer blank was spectrum was obtained followed by the spectra of apo-shPARP1-ZF1 and apo-shPARP1-ZF2, and their corresponding Zn-bound spectra (via addition of 1 equivalent ZnCl_2_). All samples were prepared with a peptide concentration of 50 µM in anaerobic10 mM Sodium Phosphate buffer, pH 7.5. Experiments were performed in triplicate.

### Preparation of hydrogen sulfide

Sodium sulfide (Na_2_S) powder was transferred into screw capped amber vials wrapped in parafilm and stored in an anaerobic chamber (3% H2/97% N2). Degassed Milli-Q water was added to the Na_2_S power to produce hydrogen sulfide (H_2_S). Samples were quantified via UV-visible absorbance at 230 nm (Ɛ = 7800 M-1 cm-1), prior to each experiment. *Warning*: H₂S is a toxic gas generated from concentrated sulfur salt stock solutions and poses significant health hazards. All H₂S solutions should be kept sealed, and concentrated stocks handled only in a fume hood. If exposure is suspected, immediately move to fresh air.

### Preparation of NBF-Cl, dimedone, HE, and SOD stocks

NBF-Cl and dimedone stocks were freshly prepared before each experiment using H_2_O as described in the reagents section. Hydroethidine (HE) was aliquoted into screw-cap amber vials in an anerobic chamber (3% H_2_/97% _N2_). DMSO was used to resuspend the master stock at 3.17 mM. 500 mL aliquots were then stored at −20 °C. Superoxide dismutase (SOD) was aliquoted into screw-cap amber vials in an anerobic chamber (3% H_2_/97% N_2_). 50 mM sodium phosphate pH 7.5 was used to resuspend the master stock at 1 mg/mL (Sigma). 500 mL aliquots were then stored at −20 °C.

### Measurement of persulfidation of PARP1 peptides generated via addition of H_2_S (with O_2_) via NBF-dimedone switch labeling assay

This procedure follows that previously reported by our laboratory [17]. Briefly, Zn(II)-shPARP1-ZF1 and Zn(II)-shPARP1-ZF2 stocks were prepared under anaerobic conditions to final concentration of 15 µM in 100 mM HEPES at pH 7.5, followed by lyopholization and re-supension in H_2_O that had been oxygenated with O_2_ gas. One equivalent of Na_2_S in H_2_S was then added with vigorous shaking followed by addition of 100 µM NBF-Cl (4-chloro-7-nitrobenzofurazan). The optical spectra were then measured every 30 s until no more changes were observed (ca. 30 min, with mixing between scans). Next, 200 µM dimedone (5,5-dimethylcyclohexane-1,3-dione) was added, mixed, and scanned every 30 s until no more changes were observed (ca. 30 min). The analogous experiments with apo-shPARP1 peptides led to precipitation. Experiments were performed in triplicate.

### Intrinsic Tryptophan and tyrosine fluorescence to measure unfolding of PARP1 peptides with O_2_ + H_2_S

Fluorescence was monitored utilizing an ISS K2 spectrofluorometer using quartz fluorometer micro cuvettes with (Starna). Excitation slit widths were 0.5 mm and the emission slit widths were 1.0 mm. The excitation wavelength was set at 280 nm while the emission wavelength was from 290 nm to 400 nm. Apo-shPARP1-ZF1 and apo-shPARP1-ZF2 were prepared under anaerobic conditions in a 100 mM HEPES pH 7.5 buffer at a final concentration of 2.5 µM (shPARP1-ZF1) and 3.2 µM (shPARP1-ZF2) and the fluorescence spectra were obtained. 1 equivalent ZnCl_2_ was then added to each peptide under anaerobic conditions and the fluorescence specra were measured. The samples were then exposed to air by opening the cuvettes on the benchtop and adding 10-fold H_2_S, mixing the solution and obtaining the fluorescence spectrum. The fluorescence intensity was monitored over time for up to 30 min mixing between each scan. Experiments were performed in triplicate.

### Observing superoxide intermediates during the reactions of PARP1 peptides with H_2_S (with O_2_) via DHE

Fluorescence was monitored utilizing an ISS K2 spectrofluorometer using quartz fluorometer micro cuvettes (Starna). Excitation slit widths were 1.0 mm and the emission slit widths at 2.0 mm. The excitation wavelength was set at 466 nm while the emission wavelength was scanned from 510 nm to 680 nm. Zn(II)-shPARP1 stocks for both shPARP1-ZF1 and shPARP1-ZF2 were prepared using 10 µM protein and 1 equivalent ZnCl_2_ with 10 mM phosphate at pH 7.5 in a Coy anaerobic glovebox. The peptide stocks were lyophilized, removed from the Coy box and the samples were resuspended on the bench top in milliQ H_2_O that had been oxygenated with O_2_ gas for at least 1 h prior. The spectra for Zn(II)-PARP1 was scanned with excitation slit widths at 0.5 mm and the emission slit widths at 1.0 mm at excitation wavelength was set at 280 nm while the emission wavelength was scanned from 310 nm to 400 nm and again with experimental parameters described above. Next, 100 µM DHE (dihydroethidium) at was added, mixed well, and scanned immediately. 1 mM Na_2_S (in H_2_O) was then added, mixed well and scanned immediately. Fluorescence spectra was measured every 15–30 min for 2 h with hand-mixing between scans, and changes were monitored over time.

### Trapping superoxide intermediates during reactions of PARP1 peptides with H_2_S (with O_2_) via SOD

Fluorescence spectra were obtained using an ISS K2 spectrofluorometer with quartz fluorometer micro cuvettes (Starna). Excitation slit widths were 1.0 mm and the emission slit widths were 2.0 mm. The excitation wavelength was set at 466 nm while the emission wavelength was scanned from 510 nm to 680 nm. Zn(II)-shPARP1 stocks for both shPARP1-ZF1 and shPARP1-ZF2 were prepared using 10 µM protein and 1 equivalent ZnCl_2_ with 10 mM phosphate at pH 7.5 in a Coy anaerobic glovebox. The stocks were lyophilized and then brought out to the bench top and the samples were resuspended in milliQ H_2_O that had been oxygenated with O_2_ gas for at least 1 h. The fluorescence spectra for Zn(II)-shPARP1-ZF1 and Zn(II)-shPARP1-ZF2 were then obtained, after which 100 µM DHE (dihydroethidium) at was added, mixed well, and the fluorescence spectra obtained. 1 mM Na_2_S (in H_2_O) was then added, mixed well and scanned immediately. 0.1 µM SOD was then added to the reaction mixture and the fluorescence spectra were measured every 15–30 min for 2 h with hand-mixing between scans. Controls in which no SOD was added were included.

## Results and discussion

### Preparation of shPARP1-ZF1 and shPARP1-ZF2 maquettes

ZF proteins are often studied as isolated domains, because they fold around zinc independently of the whole protein [69]. Several different constructs of PARP1 made up of a short peptides or maquettes that model the ZF1 and ZF2 domains have been reported previously [57]. We identified two of these constructs, corresponding to the first and second ZF domains, for this work. These constructs are short peptides, which we named shPARP1-ZF1 and shPARP1-ZF2. shPARP1-ZF1 contains 28 amino residues 17–31 and 47–60, and has the sequence GRAS**C**KK**C**SESIPKDKVP**H**WYHFS**C**FWKV and shPARP1-ZF2, which contains 28 amino acids, is made of residues 121–135 and 153–166, and has the sequence NRSTCKG**C**MEKIEKGMIDRWY**H**PG**C**FVKN. These maquettes are shorter than the full-length PARP1 ZF domains, the spacing between coordinating ligands are homologous to other known CCHC-type ZFs, and they have previously been shown to be soluble when Zn is bound, so we selected these as models of the CCHC PARP1 and related ZFs. In Table 1 the sequence of the peptides utilized here are shown, aligned with several CCHC type ZF homologs as well as the longer PARP1 peptides that have been reported (lgPARP1) [66, 67, 70]. In addition to the conserved CCHC residues, positively charged lysine residues are always found between the first and second cysteine residues. The constructs were prepared via peptide synthesis, isolated in the reduced, apo-form via reduction with TCEP, purified via reverse phase HPLC and lyophilized and stored under anerobic conditions in a Coy glovebox. The purity of constructs was confirmed by SDS-PAGE and molecular weight was confirmed by MALDI-TOF-MS.

**Table 1:**
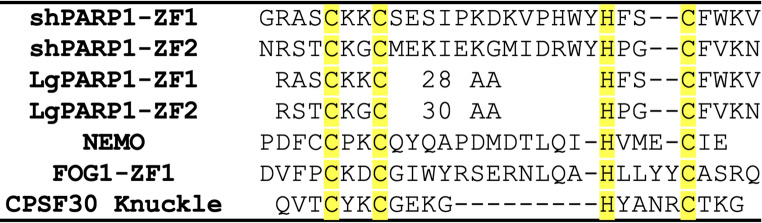
Sequence alignment of the shPARP1-ZF1 and shPARP1-ZF2 peptides, the along with several other representative CCHC-type ZF domains. Conserved zinc-binding residues are highlighted in yellow

### Co(II) and Zn(II) binding to PARP1 peptides

**Fig. 2:**
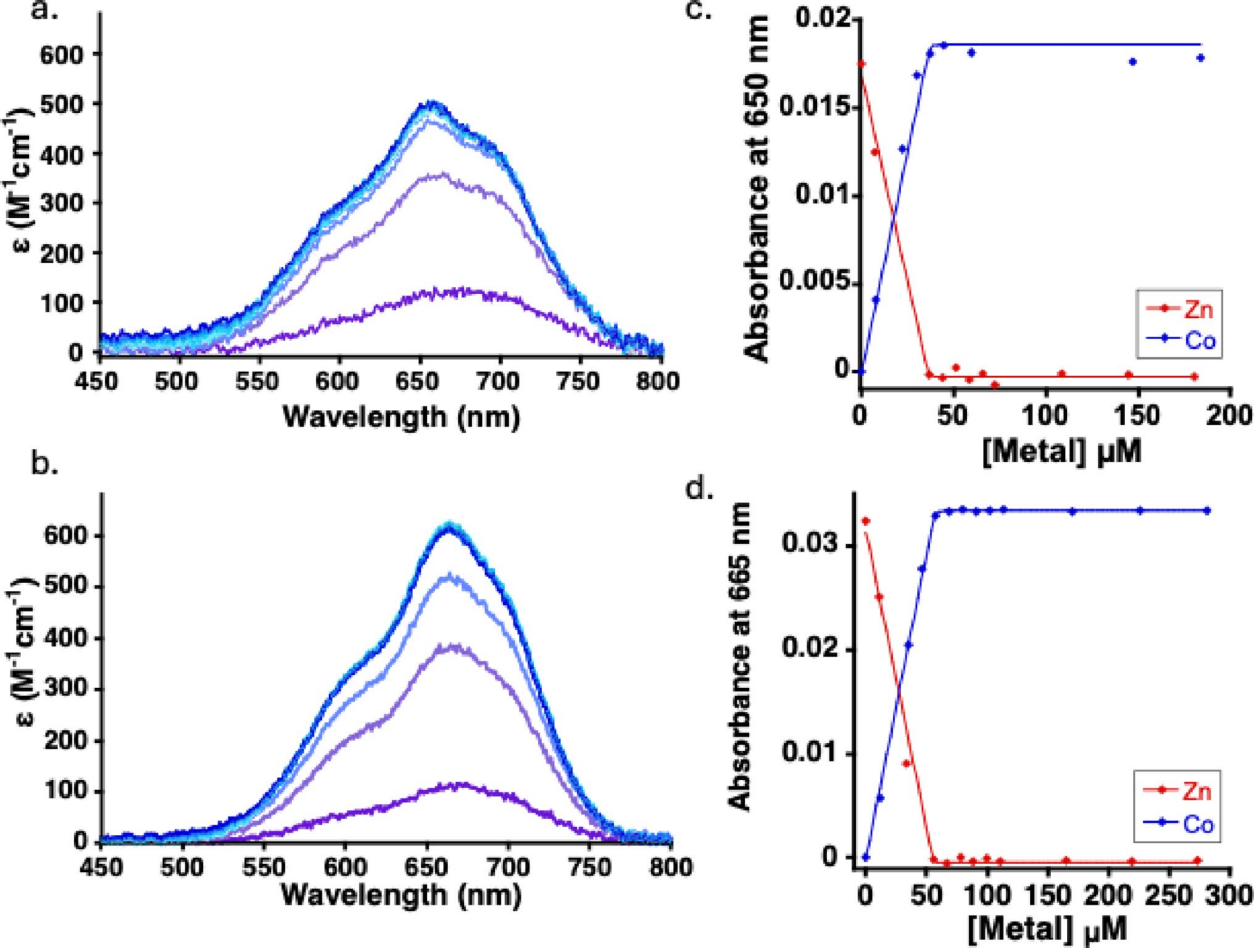
Plot of the change in the absorption spectrum between 450–800 nm as Co(II) is titrated into (**a**) apo - shPARP1-ZF1 and (**b**) apo- shPARP1-ZF2. The absorbance spectra were converted to extinction coefficients. Plot of the increase in the absorbance at 650 nm and 655 nm, respectively as CoCl_2_ is titrated with (**c**) apo-shPARP1-ZF1 and (**d**) apo-shPARP1-ZF (blue dots) up to 5 equivalents. The data are fit to a 1:1 binding model, as indicated by the solid blue lines. Plots of the decrease in absorbance at 650 and 655 nm that is observed as ZnCl_2_ is titrated with (**c**) Co-shPARP1-ZF1 and (**d**) Co-shPARP1-ZF1 (red dots) up to 5 equivalents, resulting in a decrease in the absorbance due to Zn exchanging with Co. The data are fit to a competitive binding model, as indicated by the solid red lines. All titrations were performed with in 100 mM HEPES buffer, pH 7.5

To confirm that shPARP1-ZF1 and shPARP1-ZF2 bind Zn(II) and to determine upper limit binding affinities (K_d_s), Co(II)/Zn(II) titrations were performed. Co(II) was titrated with apo-shPARP1-ZF1 and with apo-shPARP1-ZF2. Co(II) is commonly used as a spectroscopic probe for zinc binding to ZFs, as Zinc is a d^10^ metal and therefore spectroscopically silent [68, 71]. Figure 2a shows the change in the absorption spectra that was observed a Co(II) was titrated with apo-shPARP1-ZF1 or apo-shPARP1-ZF2. Upon addition of Co(II), an increase in absorbance was observed between 500 nm and 800 nm due to d-d transition bands indicative of cobalt binding [68, 72]. These data are consistent with spectra those reported by Liu and Hudson [57]. The molar absorptivity for cobalt binding to shPARP1-ZF1 was determined to be 500 M^−1^cm^−1^ at 650 nm. The molar absorptivity for cobalt binding to shPARP1-ZF2 was determined to be 600 M^−1^cm^−1^ at 665 nm. These values are typical for cobalt binding in a tetrahedral geometry and the shape of the spectra match those reported for other PARP1 peptides [73–75]. The Co(II) data were fit to a 1:1 binding equilibria, and upper limit dissociation constants, K_d_ (Co) ≤ 7.4 × 10^− 8^ M (ZF1) and K_d_ (Co) ≤ 2.4 × 10^− 8^ M (ZF2) were determined. Zinc binding was then measured using a competitive titration of ZnCl_2_ with the Co(II)-PARP1 peptides. In these experiments, Co(II)-PARP1 peptides were titrated with ZnCl_2_ resulting in the loss of d-d transition bands, indicating that zinc is replacing cobalt at the ZF binding site. This is consistent with zinc binding preferentially to tetrahedral sites over cobalt due to ligand field stabilization energy differences [68]. The data were fit to a competitive binding model and upper limit dissociation constants, K_d_ (Zn^2+^) ≤ 4.2 × 10^–12^ M (ZF1) and K_d_ (Zn^2+^) ≤ 1.2 × 10^–11^ M (ZF2) were determined (Fig. 2). Collectively, the upper limit K_d_ values for Co(II) -PARP1 and Zn(II) -PARP1 are comparable to similar reports for other ZFs [7, 8, 67, 73, 76]. We note that Hartwig and Bal have reported Co/Zn affinities for longer PARP-1 ZF peptides, and they observe tighter Zn binding to ZF2 than ZF1, which may be due to the additional amino acids within the peptide sequence affecting the thermodynamics of Zn binding [67]. These data show that the two PARP1-ZF1 and PARP1-ZF2 constructs bind cobalt and zinc in a tetrahedral geometry with upper limit dissociation constants in the ranges typically reported for ZF domains.

### Circular dichroism (CD) to measure folding of PARP1 peptides

**Fig. 3:**
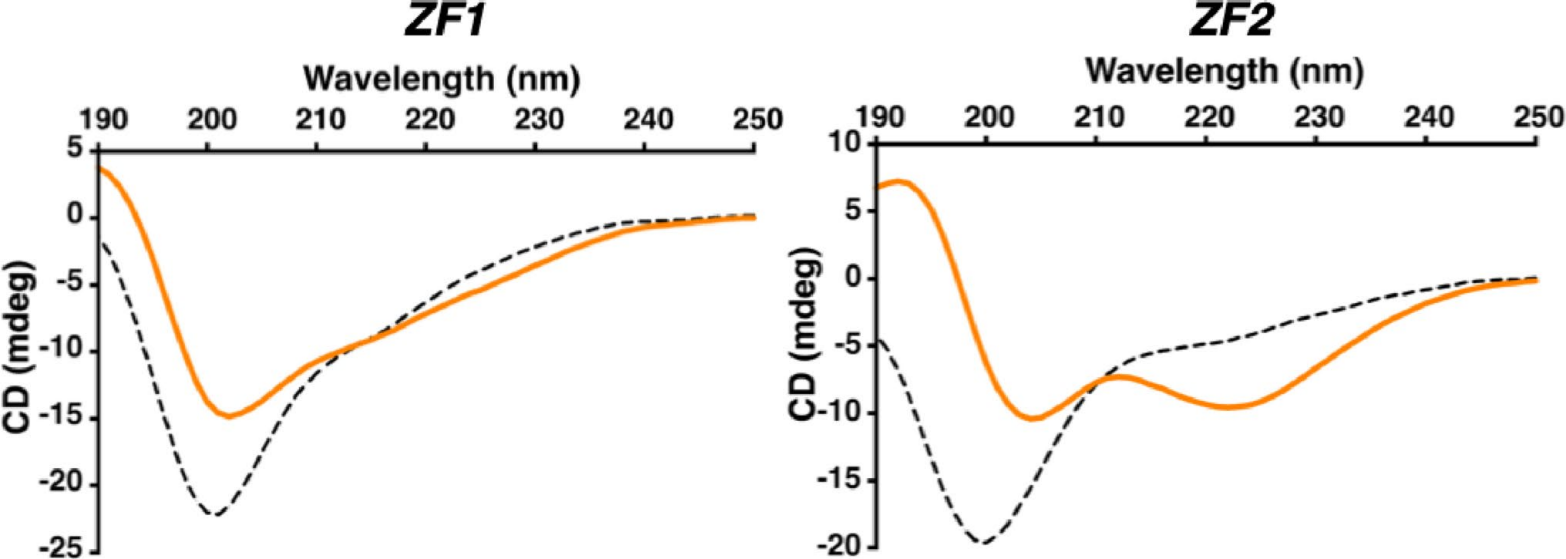
Overlay of the circular dichroism spectra for (left) apo- shPARP1-ZF1 (dotted black) and Zn(II)-shPARP1-ZF1(orange) and (right) apo-shPARP1-ZF2 (dotted black) and Zn(II)-shPARP1-ZF1 (orange) measured between 190 and 250 nm. All experiments performed at 50 $\:{\upmu\:}\mathrm{M}\:$protein in 10 mM sodium phosphate, pH 7.5 under anaerobic conditions

To determine how zinc binding affects the secondary structure of the two peptides, circular dichroism (CD) was utilized. Figure 3 shows the CD spectra of apo-PARP1 (dotted black) and Zn(II)-PARP1 (orange line) prepared under anerobic conditions for both shPARP1-ZF1 and shPARP1-ZF2. Differences in the CD spectra for apo-PARP1 compared to Zn(II)-PARP1 for both shPARP1-ZF1 and shPARP1-ZF2 were observed, with shPAPRP-ZF2 exhibiting a more pronounced change in the secondary structure upon Zn coordination. The CD data were analyzed using BeStSel and the analysis predicted the following secondary structure for each domain - shPARP1-ZF1 (48% helix, 49% antiparallel and 3% other) and shPARP1-ZF2 (54% helix, 23% antiparallel, 2% parallel, and 21% other) [77]. In a published X-ray structure of PARP1, these secondary structural features are observed and ZF2 has a longer α-helical region compared to ZF1. These CD data are consistent with this difference [47]. CD data has also been reported for longer PARP1 peptides, and similar changes have observed upon Zn binding, indicating that these secondary structural features form when Zn is bound [67].

### H_2_S reactivity of PARP1-ZF1 and PARP1-ZF2: evidence for persulfidation

**Fig. 4:**
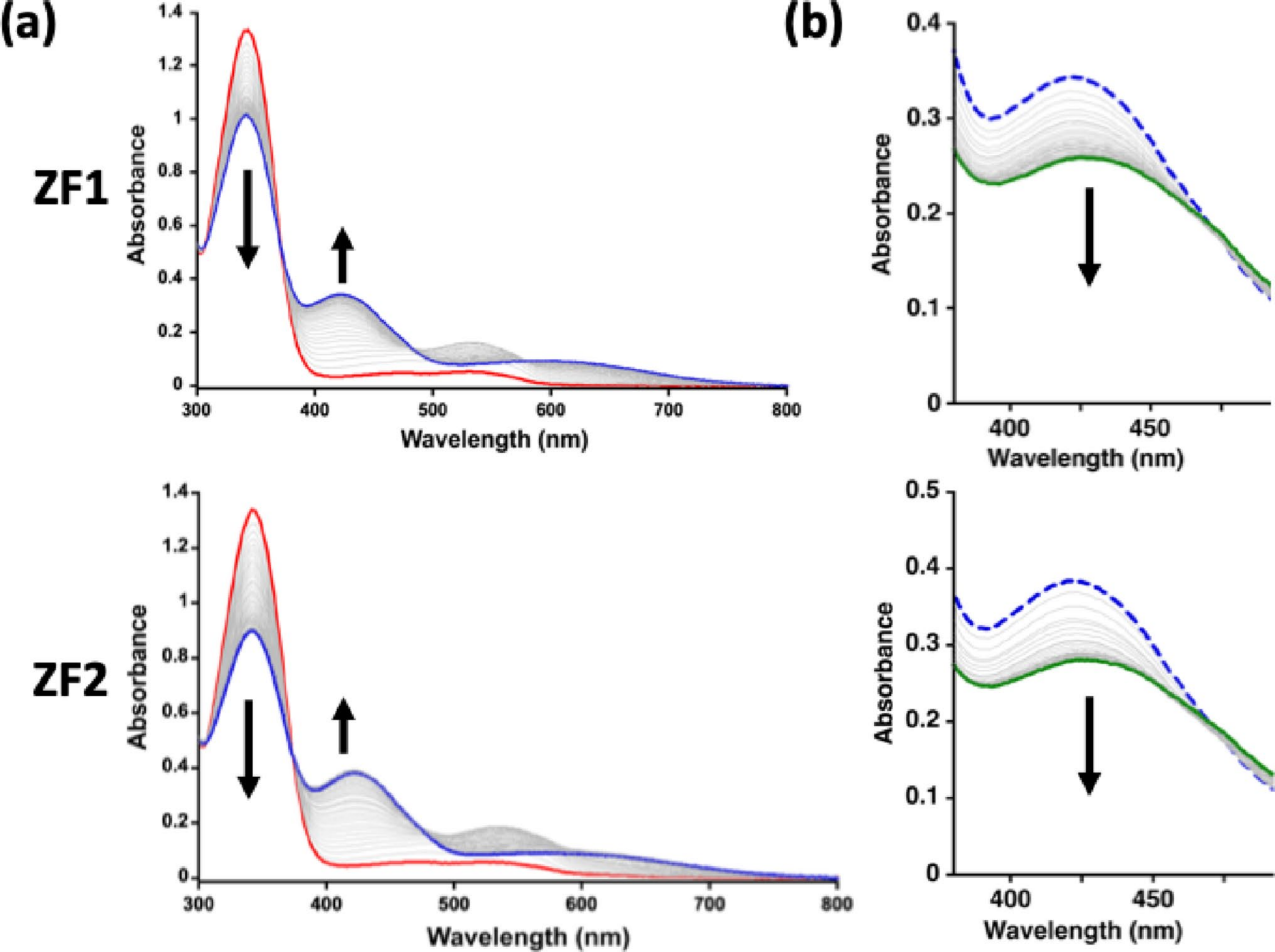
a. Plot of the change in the absorption spectra during the in situ dimedone switch experiment. Panel A: To 15 µM Zn(II)-shPARP1-ZF1 (top) or Zn(II)-shPARP1-ZF1(bottom) in oxygenated buffered, 1 equiv. H_2_S was added followed by addition of 100 µM NBF-Cl. The absorbance spectra were recorded every 30 s up to 30 min. Panel B: 200 µM dimedone was added to the NBF-labeled ZF1 and ZF2 solutions, and the absorbance spectra were recorded every 30 s up to 30 min. All experiments were performed in 100 mM HEPES, pH 7.5

To determine whether H_2_S reacts with shPARP1-ZF1 and shPARP1-ZF2, to form persulfides, a two-step persulfide selective labelling method involving NBF-Cl and dimedone was utilized [17]. In this approach, Zn(II)-PARP1 is reacted with H_2_S followed by the addition of NBF-Cl which ‘tags’ all sulfur functional groups including persulfides. Following the NBF-labeling step, dimedone is added to the reaction mixture. Dimedone selectively reacts with NBF-labeled persulfides due to electrophilicity differences and the NBF labeled persulfided ‘switches’ to a dimedone tagged sulfur [17, 33, 78]. NBF and dimedone labeling can be followed by UV-visible spectroscopy [17]. For the experiments presented here, when Zn(II)- shPARP1-ZF1 and Zn(II)-PARP1-ZF2 were reacted with H_2_S in the presence of O_2_, followed by addition of NBF-Cl, the absorbance at 350 due to NBF-Cl, decreased while the absorbance between 400–480 nm increased, indicative of NBF labeling all sulfur functional groups (thiol, persulfides, sulfenic acid, etc.) [17, 78]. The subsequent addition of dimedone resulted in loss of absorbance centered at 422 nM, indicative of dimedone switching, and persulfidation (Fig. 4). The reactions required both Zn(II) and O_2_, as no reaction occurred when either were absent, with apo-shPARP1-ZF1 and apo-PARP1-ZF2 precipitating in the presence of O_2_.

### H_2_S reactivity of PARP1-ZF1 and PARP1-ZF2: evidence for superoxide

**Fig. 5:**
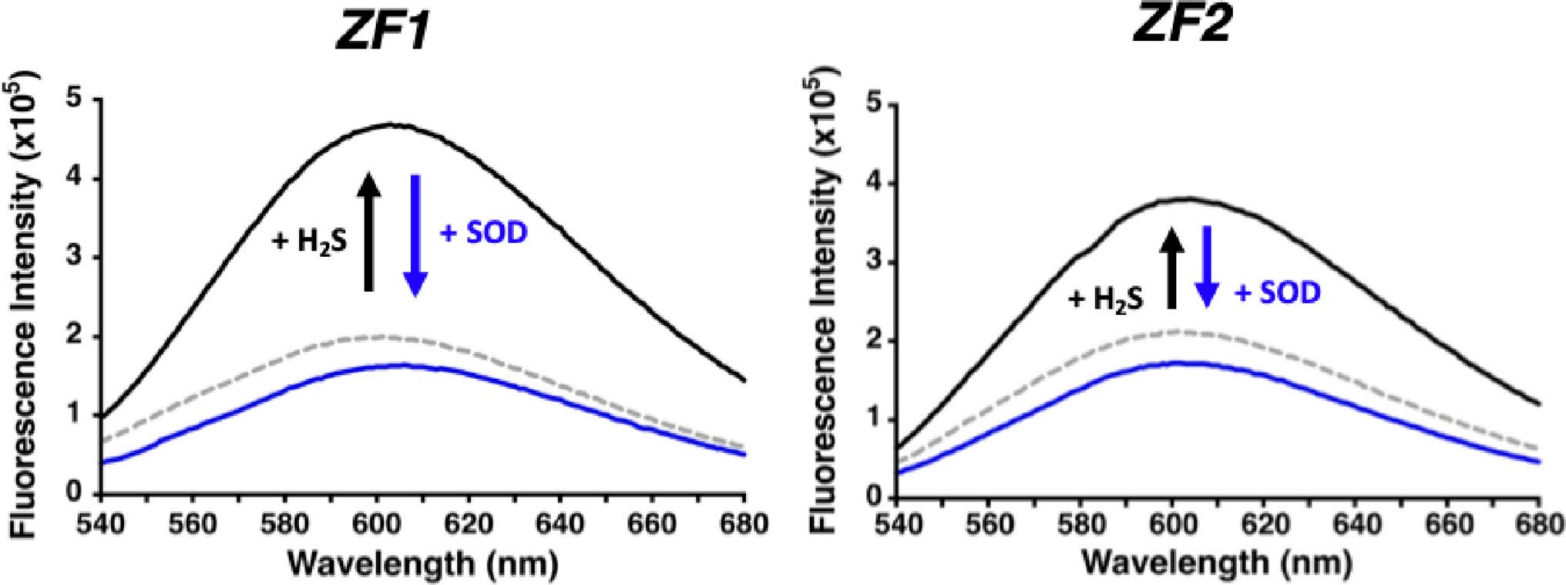
Fluorescence emission spectra of oxidized hydroethidine (black solid line) after reaction of Zn(II)-shPARP1 peptides (grey dotted lie) with H_2_S (aerobic) and HE, followed by addition of SOD (blue solid line); all experiments performed in 10 mM sodium phosphate, pH 7.5

Superoxide has been detected as an intermediate when Zn(II)-TTP and its mutants are persulfidated by H_2_S [15, 17]. We hypothesized that superoxide intermediates would also be formed when Zn(II)-PARP1 reacts with H_2_S because we observed that the PARP1 peptides, like the TTP peptides, are persulfidated in the presence of O_2_. To test this hypothesis, we utilized hydroethidine (HE) to detect superoxide intermediates [15, 17]. In this assay, Zn(II)-PARP1-ZF1 and Zn(II)-PARP1-ZF2 were each reacted with H_2_S in the presence of O_2_, followed by the addition of HE. As shown in Fig. 5, a fluorescence emission spectrum between 540 and 680 nm, indicative of HE oxidation by superoxide was observed. In addition, superoxide dismutase (SOD), which catalyzes the conversion of superoxide to oxygen, was utilized as an orthogonal method to trap superoxide. In this assay, the Zn bound peptides were reacted with H_2_S in the presence of O_2_, followed by the addition of HE then SOD. An overall decrease in fluorescence intensity was, indicating that SOD was consuming superoxide.

**Fig. 6:**
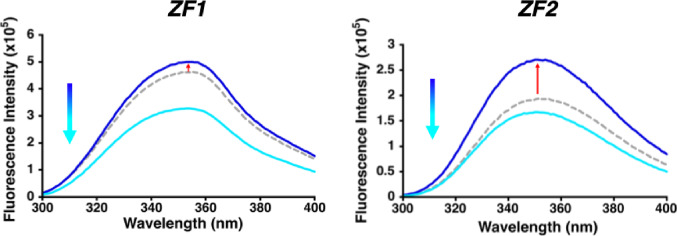
Overlay of intrinsic fluorescence spectra, left: apo-shPARP1-ZF1 (grey dashed lines), Zn(II)-shPARP1-ZF1 (dark blue line), Zn(II)-shPARP1-ZF1 + H_2_S (anaerobic, 30 min, light blue line), right: apo-shPARP1-ZF2 (grey dashed lines), Zn(II)-shPARP1-ZF2 (dark blue line), Zn(II)-shPARP1-ZF2 + H_2_S (anaerobic, 30 min, light blue line)

### Intrinsic fluorescence to assess structural changes of PARP1 persulfidation

To determine the effect of H_2_S reactivity on PARP1 structure, we utilized intrinsic tryptophan and tyrosine fluorescence. We hypothesized that persulfidation of the PARP1 ZFs would affect secondary structure. First, the fluorescence intensity for apo-PARP1-ZF1 and apo-PARP1-ZF2 were measured between 300 and 400 nm (Fig. 6, gray dashed lines). Zn was then added to each peptide, and the fluorescence intensity was again measured (Fig. 6, dark blue lines). Zn(II)-PARP1-ZF1 and Zn(II)-PARP1-ZF2 both exhibited increases in fluorescence intensity upon Zn binding. The change was larger for Zn(II)-PARP1-ZF2, which we ascribe to a larger change in secondary structure for this ZF domain, consistent with the CD changes we have observed. Zn(II)-PARP1-ZF1 and Zn(II)-PARP1-ZF2 were then each reacted with H_2_S in the presence of O_2 and_ the fluorescence spectra were obtained (light blue lines). A decrease in the fluorescence intensity was observed for both ZFs, (Fig. 6, light blue) suggesting that persulfidation disrupts the fold of each ZF.

## Conclusions

Persulfidation of ZF proteins is emerging as a new PTM; however, there are a limited number of biochemical studies that characterize the reactivity of H_2_S with isolated ZFs. These studies have focused on a CCCH type ZF that forms a structure composed of primarily loops and turns. The goal of the work described here was to examine the reactivity of a ZF with different secondary structure than TTP to determine if the same O_2_ dependent persulfidation can occur. The PARP1 ZF domains were chosen for these studies because PARP1 is found in multiple persulfide specific proteomics screens from different species and because the PARP1 ZF domains adopt secondary structure with alpha-helical and beta sheet character. We determined that the CCHC ZF peptide constructs modeling PARP1-ZF1 and PARP1-ZF2 are persulfidated by H_2_S in an O_2_ dependent manner, resulting in a loss of secondary structure. This finding parallels the results reported for TTP, in which Zn and O_2_ are both required for persulfidation, suggesting that persulfidation of ZFs occurs via a common mechanism regardless of the secondary structure. How persulfidation of PARP1 affects its biological role in DNA repair is not yet known. There is a recent report suggesting that PARP1 activity is enhanced by H_2_S [79], and future studies to link persulfidation with PARP1 mediated DNA repair activity will provide further insights into this mechanism.

## Supplementary Information

Below is the link to the electronic supplementary material.

Supplementary Material 1

## Data Availability

No datasets were generated or analysed during the current study.
