# Supplementary material for: The first and second zinc finger domains from Poly-ADP-ribose polymerase 1 (PARP1) are modified by hydrogen sulfide
Source: J Biol Inorg Chem. 2026 May 22;31(4):279–91. doi: 10.1007/s00775-026-02134-3 (PMC13215146; doi:10.1007/s00775-026-02134-3)
Supplement: Supplementary file 1 — Supplementary Material 1 [file 775_2026_2134_MOESM1_ESM.docx]

The First and Second Zinc Finger Domains from Poly-ADP-ribose polymerase 1 (PARP-1) are modified by Hydrogen Sulfide

*Ayanna J. Williams, Sarah L.J. Michel^*^*

Department of Pharmaceutical Sciences, University of Maryland Baltimore School of Pharmacy, Baltimore, MD 21201 USA

^*^To whom correspondence should be addressed, Email: [smichel@rx.umaryland.edu](mailto:smichel@rx.umaryland.edu)

ORCID: 0000-0002-6366-2453

***
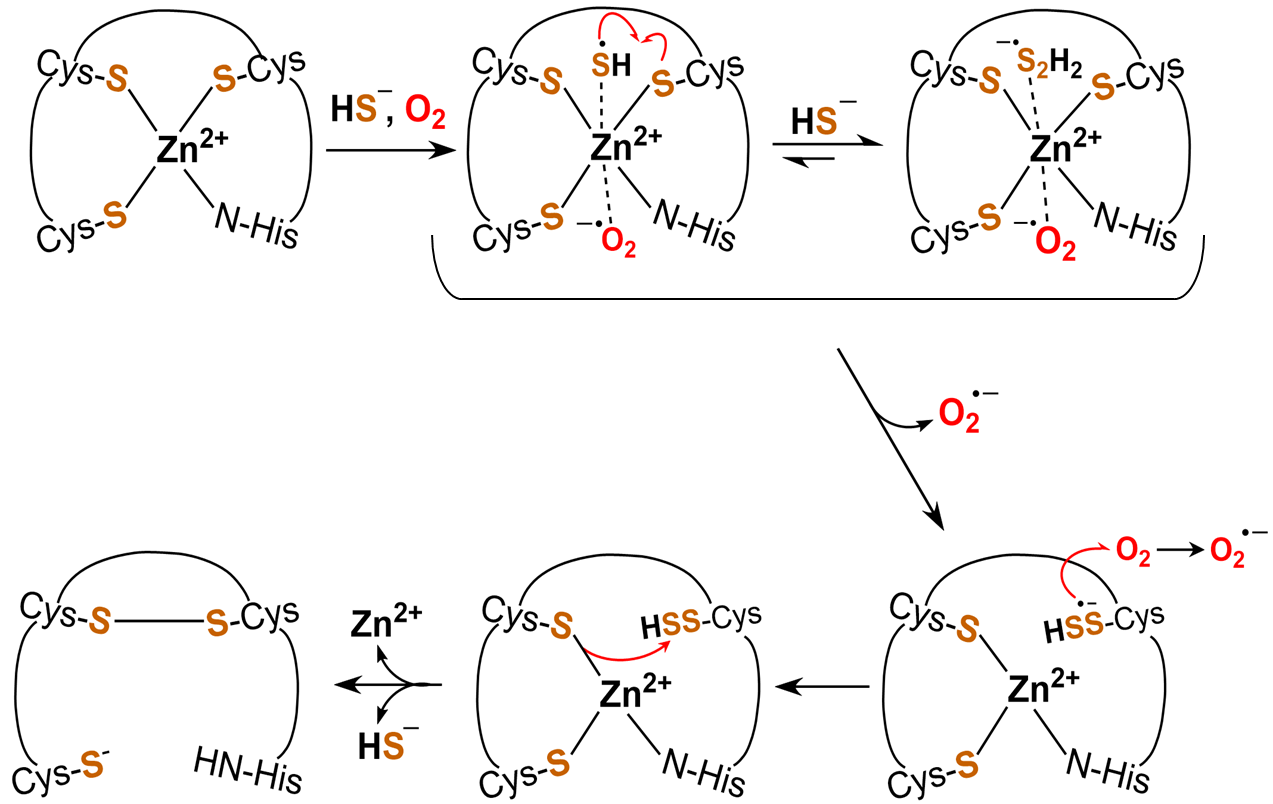
***

**Scheme S1**. Proposed mechanism for ZF. Adapted from Stoltzfus, A. T., Ballot, J. G., Vignane, T., Li, H., Worth, M. M., Muller, L., Siegler, M. A., Kane, M. A., Filipovic, M. R., Goldberg, D. P., & Michel, S. L. J. (2024). Chemoselective proteomics, zinc fingers, and a zinc(II) model for H2S mediated persulfidation. *Angew. Chem. Int. Ed. Engl.* 63:e202401003
